# Physical Activity and Diet Quality: Effects on Cardiovascular Morbidity in Women with Turner Syndrome—Results from an Online Patient Survey

**DOI:** 10.3390/jcm11010167

**Published:** 2021-12-29

**Authors:** Leonie Arnold, Martina Bacova, Robert Dalla-Pozza, Nikolaus Alexander Haas, Felix Sebastian Oberhoffer

**Affiliations:** Department of Pediatric Cardiology and Intensive Care, Medical Hospital of the University of Munich, Ludwig Maximilians University Munich, 81377 Munich, Germany; Leonie.Arnold@med.uni-muenchen.de (L.A.); Martina.Bacova@med.uni-muenchen.de (M.B.); Robert.Dallapozza@med.uni-muenchen.de (R.D.-P.); Nikolaus.Haas@med.uni-muenchen.de (N.A.H.)

**Keywords:** Turner syndrome, physical activity, diet quality, cardiovascular morbidity

## Abstract

Turner syndrome (TS) is a rare chromosomal disease with increased cardiovascular morbidity and mortality. The aim of this study was to investigate the influence of physical activity and diet quality on cardiovascular morbidity in German TS women. An anonymous online questionnaire was established. The questionnaire was based on the 2020 WHO recommendations on physical activity and sedentary behaviour and included the 14-Item Mediterranean Diet Assessment Tool. In addition, TS patients were asked about existing cardiovascular conditions. In total, 83 TS women were included in the final analysis. The achievement of <600 Metabolic Equivalent-minutes per week for recreational activities was significantly associated with the presence of arterial hypertension (*p* = 0.006). High adherence to the Mediterranean diet was achieved by only 20.5% of TS subjects and tended to be inversely associated with the presence of lipid metabolism disorders (*p* = 0.063). Only 37.3% of TS participants received nutritional counselling. Given the increased cardiovascular risk, specific counselling for lifestyle optimisation may play an important role in the management of TS. Further studies are required to evaluate the effects of regular aerobic physical training and different nutritional programs on cardiovascular morbidity in TS.

## 1. Introduction

Turner syndrome (TS) is a relatively rare X-chromosomal disease, affecting approximately one in 2500–3000 female newborns [1]. TS is associated with increased cardiovascular morbidity: compared to the general population, diabetes, arterial hypertension, dyslipidemia, obesity, congenital heart disease (CHD) (e.g., bicuspid aortic valve, coarctation of the aorta), aortic dilatation and aortic dissection are more prevalent in TS girls and women [2,3]. In addition, studies suggest increased arterial stiffness, which is considered an independent cardiovascular risk factor [4], associated with TS [5,6,7,8]. Cardiovascular disease is assumed to be the leading cause of death in TS patients, resulting in mortality that is threefold higher compared to the general population [9].

Physical activity has been shown to prevent cardiovascular disease and slow its progression if already present [10,11]. In 2020, the World Health Organization (WHO) updated its recommendations on physical activity and sedentary behaviour: adults are encouraged to implement at least 150–300 min of moderate-intensity aerobic physical activity or at least 75–150 min of vigorous-intensity aerobic physical activity, or an equivalent combination of both, per week [12]. Sedentary behaviour should be minimised [12]. Furthermore, muscle-strengthening activities including major muscle groups should be performed on at least two days per week at moderate or greater intensity [12]. While significantly reduced cardiorespiratory fitness in TS patients is assumed [13], the effects of physical activity on cardiovascular morbidity in TS is still unknown.

Besides physical activity, low diet quality has been demonstrated to be associated with increased cardiovascular risk [14]. Multiple studies suggest that the Mediterranean diet in particular is inversely associated with the presence of excess weight, arterial hypertension and diabetes [15,16,17]. In 2012, Martínez-González et al. established a 14-item tool measuring adherence to the Mediterranean diet, facilitating a rapid diet quality assessment [15]. To the best of our knowledge, the influence of diet quality on cardiovascular morbidity in TS has not yet been evaluated.

The aim of this study was to investigate the influence of physical activity and diet quality on cardiovascular morbidity in women with TS using a self-reporting questionnaire. The questionnaire was based on the 2020 WHO recommendations on physical activity and sedentary behaviour and included the 14-Item Mediterranean Diet Assessment Tool established by Martínez-González et al. [12,15].

## 2. Materials and Methods

### 2.1. Ethical Approval

The study was conducted according to the guidelines of the Declaration of Helsinki and approved by the Ethics Committee of the Ludwig Maximilians University Munich (Munich, Germany) (protocol code: 21-0403, date of approval: 7 May 2021). Prior digital informed consent was obtained from all study participants.

### 2.2. Study Design

For this study, an anonymous online questionnaire was established in German using the website SurveyMonkey (San Mateo, CA, USA). To be eligible for participation, study subjects had to digitally confirm the onset of TS and a minimum age of ≥18 years.

TS patients were recruited in cooperation with the German Turner syndrome Association (Turner-Syndrom-Vereinigung Deutschland e.V., Dornburg, Germany). In addition, national TS centers, university hospitals, pediatric cardiologists and cardiologists specialising in treating adults with congenital heart disease were informed in writing of the ongoing study. To ensure maximum coverage, German social media channels focused on TS were engaged to inform their subscribers about the ongoing survey. For this study, replies obtained between May and October 2021 were analysed.

### 2.3. Assessment of Patient Characteristics and Cardiovascular Morbidity

TS patients were asked for age (years), body height (cm) and body weight (kg). Body mass index (BMI, kg/m^2^) was subsequently calculated for each study participant. The following weight classifications were applied: underweight if BMI < 18.5 kg/m^2^, normal weight if BMI ≥ 18.5 but <25 kg/m^2^, overweight if BMI ≥ 25 but <30 kg/m^2^ and obese if BMI ≥ 30 kg/m^2^. Histories of arterial hypertension, glucose metabolism disorders, lipid metabolism disorders, CHD, heart surgery, stroke and smoking were collected. Study participants with a history of arterial hypertension, glucose metabolism disorders or lipid metabolism disorders were asked if medication was taken regularly. In addition, study participants were asked whether they had regularly seen a cardiologist and an endocrinologist. Study participants who only partially answered the questions on patient characteristics and/or cardiovascular morbidity were excluded from further analysis.

### 2.4. Assessment of Physical Activity

For the assessment of physical activity and sedentary behaviour, the German version of the Global Physical Activity Questionnaire (GPAQ), officially provided by the WHO, was applied [18]. Picture cards were presented digitally for each activity type [18]. Metabolic Equivalents (MET) were utilised to further analyse GPAQ data. One MET, equivalent to 1 kcal/kg/h, is defined as the energy spent during sedentary behaviour [18]. In comparison to sedentary behaviour (1 MET), caloric consumption is assumed to be fourfold higher when moderately active (4 METs) and eightfold higher when vigorously active (8 METs) [18]. Hence, to ensure maximal comparability of physical activity, total MET-minutes per week were calculated in accordance with the GPAQ recommendations [18]: minutes spent per week with moderate activity were multiplied by four and minutes spent per week with vigorous activity were multiplied by eight; the resulting values were summed [18]. MET-minutes per week were assessed for the following subdomains: activity at work, travel to and from places and recreational activities. Study participants met WHO recommendations on physical activity if they achieved ≥ 600 MET-minutes per week [18].

The percentage of TS subjects reporting sedentary behaviour ≤ 8 h per day was investigated [19]. TS patients were further asked whether muscle-strengthening activities at moderate or greater intensity that involve all major muscle groups were implemented on at least two days per week [12]. In addition, the effect of the coronavirus pandemic on physical activity was studied. Data on physical activity were cleaned according to GPAQ recommendations [18]. Study participants who only partially answered the questions on physical activity were excluded from further analysis.

### 2.5. Assessment of Diet Quality

For assessment of diet quality, the validated 14-Item Mediterranean Diet Assessment Tool established by Martínez-González et al. was translated into German and utilised [15]. In accordance with Martínez-González et al., a score ≤ 7 points was considered low adherence and a score > 7 points high adherence to the Mediterranean diet [15]. Since study participants rarely achieved a score ≥ 10 points, a division into a third category was not practical. TS patients were asked whether their treating physician notified them of the importance of healthy lifestyle habits as well as if they participated in nutritional counselling and whether they knowingly implemented a healthy lifestyle in their daily routine. Moreover, the effect of the coronavirus pandemic on diet quality was evaluated.

Study participants who only partially answered the questions on diet quality were excluded from further analysis.

### 2.6. Statistical Analysis

Continuous variables were tested for normal distribution by evaluating histograms, QQ-plots, the Kolmogorov–Smirnov test, and the Shapiro–Wilk test. Means and standard deviations were calculated for all continuous variables. Percentages and counts were measured for ordinal and nominal variables. Adherence to the 2020 WHO recommendations on physical activity and sedentary behaviour and adherence to the Mediterranean diet were tested against arterial hypertension, glucose metabolism disorders, lipid metabolism disorders, CHD and stroke using a chi-squared or Fisher’s exact test for counts smaller than five. “Being notified by the treating physician on the importance of healthy lifestyle habits”, “receiving nutritional counselling” and “knowingly implementing healthy lifestyle habits in the daily routine” were tested against adherence to the Mediterranean diet using a chi-squared test. The influence of MET-minutes per week of recreational activities on weight classification was tested using the *t*-test for unpaired data. A *p*-value of <0.05 was considered statistically significant. All analyses were conducted using SPSS (IBM SPSS Statistics for Windows, version 26.0. IBM Corp., Armonk, NY, USA).

## 3. Results

### 3.1. Patient Characteristics and Cardiovascular Morbidity

In total, 105 TS women participated in the online survey. Of these, 22 were excluded due to incomplete responses. Eighty-three TS patients were included in the final analysis. Information on patient characteristics and cardiovascular morbidity is presented in Table 1. Point-biserial correlation revealed no significant correlation between BMI and the presence of arterial hypertension (r = −0.218, *p* = 0.059), glucose metabolism disorders (r = 0.172, *p* = 0.127) or lipid metabolism disorders (r = 0.008, *p* = 0.947).

### 3.2. Physical Activity and Cardiovascular Morbidity

“Moderate physical activity at work” was reported by 51.8% of study participants with an average of 590.84 ± 789.21 min per week, while “intense physical activity at work” was reported by 14.5% of TS women with an average of 139.16 ± 482.0 min per week. “Travel to and from places” was documented by 81.9% of the participants with an average of 313.04 ± 408.41 min per week. Among study participants, 79.5% and 34.9% engaged in moderate and intense recreational physical activities with an average of 141.27 ± 164.19 and 60.96 ± 146.73 min per week, respectively. When asked about muscle-strengthening activities at moderate or greater intensity that involve all major muscle groups, 21.7% of participants answered yes, with an average of 2.94 ± 2.23 workouts per week. 66.3% of study participants reported sedentary behaviour ≤8 h per day. The average time spent on sedentary behaviour was reported to be 6.81 ± 2.95 h per day. Table 2 summarises information on physical activity measured in MET-minutes per week and the corresponding percentage of TS women meeting the WHO guidelines on physical activity of ≥600 MET-minutes per week.

**Figure 1:**
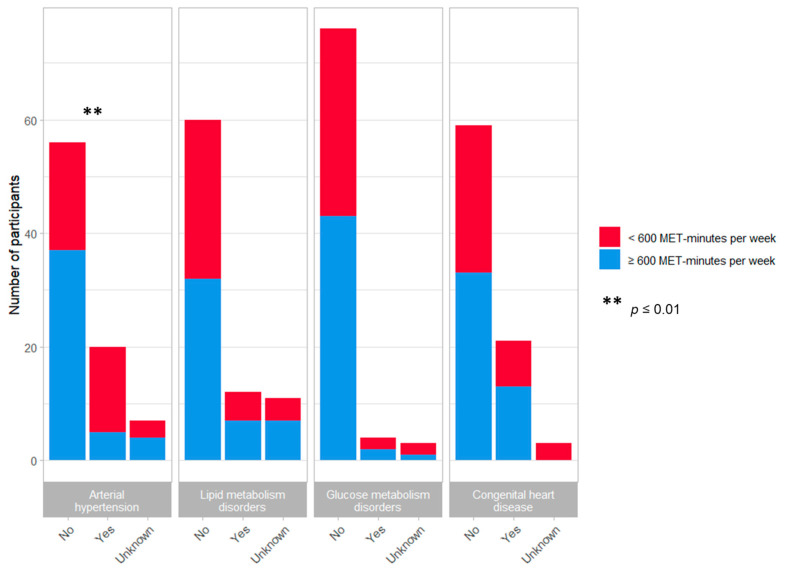
MET-minutes per week for recreational activities and cardiovascular morbidity in Turner syndrome patients. Chi-squared tests or Fisher’s exact test for counts smaller than five were applied to test for significance. ** *p* ≤ 0.01.

**Figure 2:**
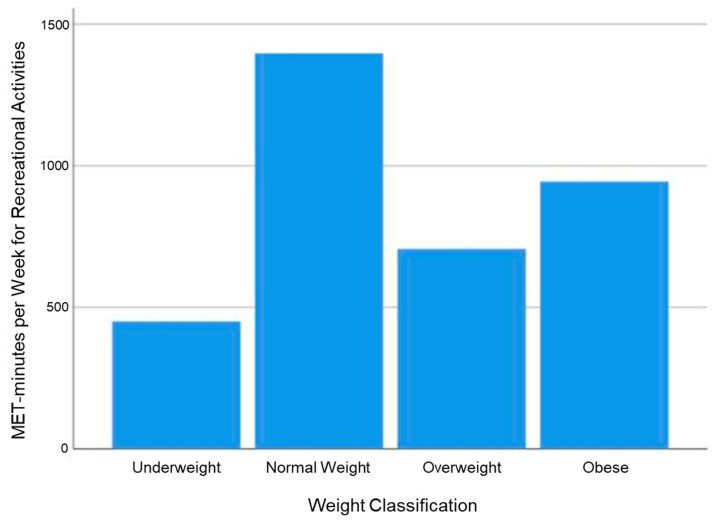
MET-minutes per week for recreational activities relative to weight classification of Turner syndrome patients. Chi-squared tests or Fisher’s exact test for counts smaller than five were applied to test for significance.

The achievement of only < 600 MET-minutes per week for recreational activities was significantly associated with the presence of arterial hypertension (*p* = 0.006), even when patients with CHD were excluded from analysis (*p* = 0.02), but not with the presence of lipid metabolism disorders (*p* = 0.881), glucose metabolism disorders (*p* = 0.717), CHD (*p* = 0.143), or stroke (*p* = 1.000) (Figure 1). In addition, no significant differences were demonstrated between average MET-minutes per week for recreational activities and weight classification (Figure 2).

When asked about the influence of the coronavirus pandemic on physical activity, 34.9% of TS subjects reported being less active, 45.8% being similarly active and 16.9% being more active, while 2.4% of respondents were not sure whether the coronavirus pandemic influenced their physical activity.

### 3.3. Diet Quality and Cardiovascular Morbidity

Criteria for adherence to the Mediterranean diet and the percentage of TS subjects meeting each criterion are reported in Table 3.

Participants reached a mean score of 5.92 ± 2.30 points. High adherence to the Mediterranean diet, defined as a score > 7 points, was achieved by only 20.5% of TS subjects.

**Figure 3:**
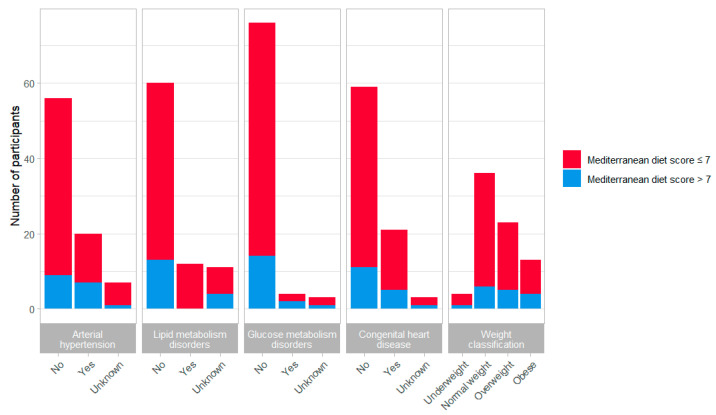
Low (score ≤ 7) and high (score > 7) adherence to the Mediterranean diet relative to cardiovascular morbidity in Turner syndrome patients. Chi-squared tests or Fisher’s exact test for counts smaller than five were applied to test for significance.

The influence of high adherence to the Mediterranean diet on cardiovascular morbidity was not significant for arterial hypertension (*p* = 0.197), glucose metabolism disorders (*p* = 0.189) and stroke (*p* = 1.000). High adherence to the Mediterranean diet tended to be inversely associated with the presence of lipid metabolism disorders but did not reach statistical significance (*p* = 0.063) (Figure 3).

Only 26.5% of TS subjects were notified by their treating physician of the importance of healthy lifestyle habits, 37.3% of participants received nutritional counselling and 84.3% of TS subjects reported knowingly implementing a healthy lifestyle in their daily routine. However, these factors were not significantly associated with high adherence to the Mediterranean diet. When asked about the influence of the coronavirus pandemic on eating habits, 21.7% of TS subjects reported having unhealthier eating habits, 55.4% similar eating habits and 19.3% healthier eating habits, while 3.6% of respondents were not sure whether the coronavirus pandemic influenced their eating habits.

## 4. Discussion

To the best of our knowledge, this is the first study that investigated the influence of physical activity and diet quality on cardiovascular morbidity in TS women. In total 83 women were included in the present study, making it one of the largest surveys of German-speaking TS subjects.

### 4.1. Association between Physical Activity and Cardiovascular Morbidity

In total, 94% of TS patients fulfilled the WHO recommendations for physical activity, which seems quite high, considering that only 38.8% of German women between the age of 30 to 44 years meet the WHO recommendations [20]. The rather high percentage of TS patients fulfilling the WHO recommendations for physical activity could potentially be affected by the rather subjective data collection from study participants. Approximately two-thirds of TS subjects displayed sedentary behaviour of ≤8 h per day. When looking at recreational physical activities, 55.4% of study participants reached a value ≥ 600 MET-minutes per week. Interestingly, the time spent with recreational physical activities correlated inversely with the presence of arterial hypertension, even when TS subjects with CHD (e.g., coarctation of the aorta) were excluded. Aggressive management of arterial hypertension is crucial for the cardiovascular outcomes of TS patients, particularly for the prevention of aortic dilatation and dissection [21,22]. In the literature, the prevalence of arterial hypertension ranges up to 60% with TS, idiopathic arterial hypertension being the most common form [2,23]. With a prevalence of 24.1%, this study might underestimate the “real” prevalence of arterial hypertension within the questioned TS women due to its subjective nature.

Excess weight was present in 49.4% of TS women. The TS prevalence of overweight and obesity was therefore distinctly higher compared to 38% of the female German population aged 30 to 39 years [24]. Considering the already increased cardiovascular morbidity, the elevated rates of overweight and obesity in a relatively young TS population demonstrated in this study are alarming. TS-specific hormonal imbalances can be named as one reason for the increased prevalence of excess weight [25]. Within this study, time spent with recreational physical activities was inversely associated with the presence of excess weight, but did not reach statistical significance. Interestingly, underweight TS subjects reported being less engaged in recreational activities compared to normal-weight peers. Within the general population, underweight is associated with lower cardiorespiratory fitness and increased overall mortality [26,27]. As this study only evaluated physical activity, further research is required to evaluate whether underweight and overweight/obese TS subjects display decreased cardiorespiratory fitness and increased mortality compared to normal-weight peers.

TS research has primarily focused on intrinsic factors causing vascular dysfunction and hence the presence of arterial hypertension. However, little is known regarding the influence of healthy lifestyle habits on vascular morbidity and excess weight in this cohort. The results of this study suggest that recreational physical activities in particular help in the prevention and management of arterial hypertension and excess weight in TS patients. Further studies are required to investigate the effects of regular aerobic physical training on vascular function and body weight management in TS.

### 4.2. Association between Diet Quality and Cardiovascular Morbidity

For the assessment of diet quality, the 14-Item Mediterranean Diet Assessment Tool established by Martínez-González et al. was applied [15]. With a mean of 5.92 points, adherence to the Mediterranean diet was rather low within the TS cohort [15]. Several studies suggest a low adherence to the Mediterranean diet to be a risk factor in the development of cardiovascular disease [15,16,17]. Lipid metabolism disorders are common in TS, and up to 50% of women have elevated total cholesterol levels [2,3]. In this study, lipid metabolism disorders were reported by 14.5% of TS patients. Besides intrinsic factors, lower estrogen levels found in TS women due to gonadal dysgenesis might partially explain the increased prevalence of lipid metabolism disorders compared to the general population [28,29]. Pirgon et al. demonstrated a significant correlation between altered cholesterol levels and carotid artery intima media thickness in young TS subjects, underlining the negative effects of dyslipidemia on the vascular system in TS [30]. A low adherence to the Mediterranean diet, suggesting a low diet quality, tended to be associated with the presence of lipid metabolism disorders in participating TS women.

To the best of our knowledge, this is the first study that investigated the effect of diet quality on cardiovascular morbidity in TS. This study indicates that an increased adherence to the Mediterranean diet might help in the management of lipid metabolism disorders. Further studies are needed to evaluate the effects of different nutritional programs on cardiovascular morbidity in TS.

### 4.3. Healthy Behaviour Counselling

Counselling on healthy nutrition and physical activity plays an important role in the cardiovascular management of TS women and should begin in early childhood, according to the clinical practice guidelines for the care of girls and women with Turner syndrome [2]. The results of this study demonstrate an alarming lack of adequate cardiovascular care among German TS women: only 57.8% of participating TS women reported being regularly seen by a cardiologist and 59% by an endocrinologist. Only 26.5% of TS subjects were notified by their treating physicians regarding the importance of healthy lifestyle habits and only 37.3% of TS participants received professional nutritional advice. When asked for the presence of cardiovascular diseases, 8.4% of TS patients stated unknown for arterial hypertension and 13.3% for lipid metabolism disorders. This suggests that approximately one in ten TS women are not familiar with crucial cardiovascular risk factors that require regular screening [2].

TS requires regular cardiological and endocrinological care, independently of cardiovascular morbidity [2]: body weight, BMI and blood pressure measurement should be performed at each clinical visit [2]. Moreover, HbA1c should be assessed annually in all adult TS subjects [2]. The evaluation of blood lipids is recommended annually in adult TS patients displaying at least one cardiovascular risk factor (e.g., arterial hypertension, excess weight, smoking, diabetes, and physical inactivity) [2]. In TS adults without structural heart disease, aortic dilatation, or arterial hypertension, transthoracic echocardiographic or cardiac magnetic imaging should be performed at least once every five to ten years by a cardiologist [2]. Cardiac monitoring might be required more frequently if one or more of the above-mentioned cardiovascular diseases is present [2]. When counselling TS patients on sport participation, aortic valve function and the presence of CHD, arterial hypertension and aortic dilatation should be considered [2]. While TS girls and women with normal aortic size are encouraged to participate in all sports, subjects with moderately to severely dilated aorta are discouraged from competitive sport participation and intense weight training [2]. To minimise adverse events due to inappropriate sport participation, prior cardiovascular screening and exercise consultation should be performed. Since the cardiovascular morbidity of TS can be complex, patients are recommended to attend specialised multidisciplinary clinics to ensure appropriate health surveillance [2].

### 4.4. Potential Influence of the Coronavirus Pandemic

The potential influence of the coronavirus pandemic on physical activity and the diet quality of study participants was considered. Approximately 35% of TS subjects reported being less physically active and 22% having unhealthier eating habits due to the coronavirus pandemic. Conversely, 16.9% of TS patients reported being more physically active and 19.3% having healthier eating habits compared to before the coronavirus pandemic. Hence, the results of this study could be possibly influenced by the current coronavirus pandemic and must be confirmed in the future.

### 4.5. Limitations

The relatively small number of TS women included in this study might be considered one limitation. However, with a sample size of 83, this study represents one of the largest surveys of German-speaking TS women. To further evaluate the effect of physical activity and diet quality on specific cardiovascular diseases in TS, international multi-center studies with a greater sample size are required. The GPAQ was established for face-to-face interviews with the opportunity for further inquiry in cases of doubtful reports [18]. This might partially explain the great amount of TS patients fulfilling the WHO recommendations on physical activity. The 14-Item Mediterranean Diet Assessment Tool established by Martínez-González et al. was translated into German, potentially altering the questions’ reception [15]. Moreover, this study used a self-reporting questionnaire. The presented data could potentially be skewed due to subjective information from study participants (e.g., only one of 12 TS subjects with dyslipidemia reported to be on lipid-lowering therapy). This study did not ask for specific TS karyotypes. As some TS karyotypes are more associated with particular cardiovascular diseases [31], the results demonstrated in this study could be altered due to potential genotype–phenotype relationships. Furthermore, study participants were not asked to report the specific type of medication taken, nor to report history of growth hormone or estrogen–progesterone therapy. In addition, study participants were only asked for the presence of, but not specific types of, glucose metabolism disorder, lipid metabolism disorder, CHD, or heart surgery. As this was an online survey for patients only, the above-mentioned questions were considered to potentially “overwhelm” some study participants and therefore lead to altered study results. In conclusion, more objective TS studies are needed that assess health behaviour through face-to-face interviews and evaluate cardiovascular morbidity and medication more precisely via medical records.

## 5. Conclusions

In this study, data from a self-administered online patient survey demonstrated that time spent on recreational physical activities was inversely associated with the presence of arterial hypertension and excess weight in TS. Adherence to the Mediterranean diet was rather low within the TS cohort. A low adherence to the Mediterranean diet tended to be associated with lipid metabolism disorders. Physical activity and diet quality play an important role in the cardiovascular management of TS girls and women. A lack of adequate cardiovascular care among German TS women was demonstrated. The results of this study could be possibly influenced by the current coronavirus pandemic and must be confirmed in the future. In addition, further studies are needed to evaluate the effects of regular aerobic physical training and different nutritional programs on cardiovascular morbidity in TS.

## Figures and Tables

**Table 1:** Patient characteristics and cardiovascular morbidity of Turner syndrome patients.

Patient Characteristics	
N	83
Age (years)	37.48 ± 11.22
Height (cm)	154.37 ± 7.04
Body Weight (kg)	62.06 ± 15.37
BMI (kg/m^2^)	25.97 ± 6.00
Underweight (N (%))	4 (4.8)
Normal weight (N (%))	38 (45.8)
Overweight (N (%))	26 (31.3)
Obese (N (%))	15 (18.1)
Arterial hypertension (N (%))	20 (24.1)
Drug therapy (N (%))	15 (18.1)
Glucose metabolism disorders (N (%))	4 (4.8)
Drug therapy (N (%))	4 (4.8)
Lipid metabolism disorders (N (%))	12 (14.5)
Drug therapy (N (%))	1 (1.2)
Congenital heart disease (N (%))	21 (25.3)
Cardiac surgery (N (%))	11 (13.3)
Stroke (N (%))	2 (2.4)
Smoker (N (%))	4 (4.8)
Regular cardiological care (N (%))	48 (57.8)
Regular endocrinological care (N (%))	49 (59.0)

Mean ± standard deviation is used for normally distributed variables. BMI, body mass index. When asked for the presence of cardiovascular disease, 8.4% of TS patients stated unknown for arterial hypertension, 3.6% for glucose metabolism disorders, 13.3% for lipid metabolism disorders and 3.6% for congenital heart disease.

**Table 2:** Physical activity and percentage of Turner syndrome patients meeting WHO guidelines.

	MET-Minutes per Week (Mean ± SD)	WHO Recommendations Met N (%)
Total physical activity	5785.54 ± 6228.57	78 (94.0)
Activity at work	3476.63 ± 5525.10	41 (49.4)
Travel to and from places	1256.14 ± 1633.64	53 (63.9)
Recreational activities	1052.77 ± 660.00	46 (55.4)

Mean ± standard deviation is used for normally distributed variables. Physical activity was measured in Metabolic Equivalent (MET)-minutes per week. WHO recommendations were met if MET-minutes per week were ≥600.

**Table 3:** Adherence to the Mediterranean diet among participating Turner syndrome women.

Questions	Criteria for 1 Point	Point Reached (%)
1. Do you use olive oil as main culinary fat?	Yes	45.8
2. How much olive oil do you consume in a given day (including oil used for frying, salads, out-of-house meals, etc.)?	≥4 tbsp	14.5
3. How many vegetable servings do you consume per day? (1 serving: 200 g (consider side dishes as half serving))	≥2 (≥1 portion raw or as a salad)	38.6
4. How many fruit units (including natural fruit juices) do you consume per day?	≥3	30.1
5. How many servings of red meat, hamburger, or meat products (ham, sausage, etc.) do you consume per day? (1 serving: 100–150 g)	<1	63.9
6. How many servings of butter, margarine, or cream do you consume per day (1 serving: 12 g)?	<1	72.3
7. How many sweet or carbonated beverages do you drink per day?	<1	61.4
8. How much wine do you drink per week?	≥7 glasses	4.8
9. How many servings of legumes do you consume per week (1 serving: 150 g)?	≥3	13.3
10. How many servings of fish or shellfish do you consume per week? (1 serving: 100–150 g of fish or 4–5 units or 200 g of shellfish)	≥3	21.7
11. How many times per week do you consume commercial sweets or pastries (not homemade), such as cakes, cookies, biscuits, or custard?	<3	73.5
12. How many servings of nuts (including peanuts) do you consume per week? (1 serving: 30 g)	≥3	28.9
13. Do you preferentially consume chicken, turkey, or rabbit meat instead of veal, pork, hamburger, or sausage?	Yes	67.5
14. How many times per week do you consume vegetables, pasta, rice, or other dishes seasoned with sofrito (sauce made with tomato and onion, leek, or garlic and simmered with olive oil)?	≥2	56.6

Questions were based on the 14-Item Mediterranean Diet Assessment Tool established by Martínez-González et al. [15].

## Data Availability

Data are available from the authors upon reasonable request.
